# ABCG Transporters in the Adaptation of Rice to Salt Stresses

**DOI:** 10.3390/ijms251910724

**Published:** 2024-10-05

**Authors:** Dan Zhang, Yuanyi Hu, Li Tang, Yaxi Du, Ruihua Mao, Xiabing Sheng, Huimin Liu, Xiaolin Liu, Bingran Zhao, Dongyang Lei

**Affiliations:** 1 College of Agronomy, Hunan Agricultural University, Changsha 410128, China; zhangdan0903@stu.hunau.edu.cn; 2 State Key Laboratory of Hybrid Rice, Hunan Hybrid Rice Research Center, Changsha 410125, China; huyuanyi@hhrrc.ac.cn (Y.H.); tangli@hhrrc.ac.cn (L.T.); shengxiabing@hhrrc.ac.cn (X.S.); liuhuimin@hhrrc.ac.cn (H.L.); liuxiaolin@hhrrc.ac.cn (X.L.); 3 National Center of Technology Innovation for Salin-Alkali Tolerant Rice, Sanya 572000, China; 4 School of Tropical Agriculture and Forestry, Hainan University, Haikou 570228, China; 22220951310156@hainanu.edu.cn (Y.D.); 23220951310204@hainanu.edu.cn (R.M.)

**Keywords:** rice, ABCG transporter, salt stress response, yeast expression

## Abstract

The ATP-binding cassette (ABC) proteins are a diverse family of transmembrane transporter proteins widely identified in various organisms. The ABCG transporters belong to the G subfamily of the ABC transporter family. Rarely research on ABCG transporters involved in salt tolerance of rice was found. In this study, the evolutionary relationships, conserved motifs, intra- and inter-species homologous genes, and cis-acting elements of ABCG subfamily members were analyzed, and the expression changes of these genes under salt stress at 0 h, 3 h, and 24 h were detected. Based on these results, the candidate gene *OsABCG7*, which is induced by salt stress, was selected for further studies. Yeast experiments confirmed that the *OsABCG7* gene might be involved in the regulation of salt tolerance. The abcg7 mutant showed a higher degree of leaf wilting and a lower survival rate, exhibiting a salt-sensitive phenotype. Systematic analysis of this family in rice helps design effective functional analysis strategies and provides data support for understanding the role of ABCG transporters under salt stress.

## 1. Introduction

Environmental stresses such as drought, flooding, high temperature, low temperature, and high salinity significantly constrain plant growth and development and lead to yield reduction, particularly for major cereal crops. Improving the stress tolerance of crops to ensure stable yields under adverse conditions is, therefore, an urgent task [1].

The impact of high salinity on plants occurs in two distinct phases: the first is the osmotic phase, where salt in the solution reduces water potential, impeding root water uptake and causing water deficit [2]; the second is the ion-specific phase, characterized by the accumulation of toxic concentrations of sodium [3]. In response to salt stress, plants have evolved a range of biochemical strategies.

To mitigate osmotic stress, plants can maintain water uptake, prevent water loss, and engage in osmotic adjustment [4]. To combat ion toxicity, plants can selectively accumulate or exclude ions, regulate root ion uptake and transport to leaves, and compartmentalize ions within cells and across entire leaves to enhance salt tolerance [5]. Salt tolerance is a multifaceted phenomenon that potentially involves various mechanisms and pathways. The cultivation of salt-tolerant rice varieties has a history of over 70 years [6]. Traditional breeding methods can acquire salt-tolerant varieties by collecting and screening a wide range of germplasm resources. Additionally, salt-tolerant varieties can be developed through hybridization, mutagenesis, and molecular marker techniques [7,8,9].

Advances in information and technology related to genetics and genomics have provided insight into the mechanisms of salt tolerance and the manipulation of plant genomes to achieve breeding goals [10]. Bioinformatics offers powerful analytical tools and methods for genetics and genomics, enabling scientists to extract valuable information from massive volumes of biological data. Researchers have utilized bioinformatics to analyze the functions and regulatory mechanisms of the *DnaJA* gene family in soybean, identifying *GmDnaJA6* as a candidate regulator of salt and alkali stress tolerance; it interacts with *GmHSP70* and may modulate salt tolerance by preventing the degradation of POD, SOD, and CAT enzymes under salt stress [11]. In sorghum, there are 210 MYB transcription factors. Through bioinformatics and transcriptomic analyses, researchers have identified a gene, *SbMYBAS1*, that negatively regulates sorghum salt tolerance and conducted functional analyses, which are of significant importance for further enhancing sorghum’s salt tolerance [12].

The ABC transporter family in plants constitutes a large protein family involved in the import and export of various metabolites within the plant cell system [13]. A core unit of ABC proteins includes a highly conserved nucleotide-binding domain (NBD) and a less conserved transmembrane domain (TMD). The NBD binds and hydrolyzes ATP, providing the energy required for transport, while the TMD is involved in substrate recognition and can form a channel for substrate translocation [14]. Half-size ABC proteins contain only one core unit, whereas full-size ABC proteins have two or more core units [15]. The ABC transporter family is currently classified into eight subfamilies (A-G and I), with no ABCH transporters found in plants [14]. The largest ABCG subfamily comprises 43 members in Arabidopsis, 51 in pigeon pea, 51 in maize, and 52 members in rice [16,17,18]. Rice ABCG proteins can be categorized into two subfamilies: the Pleiotropic drug resistance (PDR)subfamily with full-size protein (NBD-TMD)_2_ and the White-brown complex (WBC)subfamily with half-size protein NBD-TMD. Half-size ABC transporters must form homodimers or heterodimers to function effectively.

The ABCG subfamily proteins play a crucial role in the physiological and developmental processes of plants, and their functions under heavy metal, hormone, flood, and drought stress have been well described. In 2019, researchers identified *OsABCG45/PDR1*, *OsABCG36/PDR9* (abiotic stress), and *OsABCG48/PDR3*, *OsABCG53/PDR13* (biotic stress) genes as candidate genes for developing tolerance to abiotic and biotic stresses [19]. In Arabidopsis, the balance of ABA is regulated by ABCG17 and ABCG18; under abiotic stress conditions, the transcription of ABCG17 and ABCG18 is inhibited, thereby promoting the active movement and response of ABA [20]. Moreover, two ABCG proteins, *ABCG33* and *ABCG37*, were found to act as Cs+ efflux carriers, providing new data support for the detoxification of toxic metals in plants [21]. *OsPDR20* is induced by cadmium stress in rice roots, shoots, and many other organs or tissues, actively participating in cadmium accumulation and balance in rice crops [22]. Research has introduced the concept of CK (Cytokinin) transport cycling and systematically elucidated that *ABCG14* is responsible for the root assembly, aerial tissue distribution, and root unloading of CK [23]. Another study revealed that *ABCG5* can form intercellular air channels in developing seedlings, restricting light transmission in the hypocotyl, thereby contributing to the plant’s precise phototropic response [24]. Recent studies have shown that the ABCG transporters *ABCG16* and *ABCG25* are involved in the dual transport activities of ABA-GE and ABA, thereby participating in ABA-mediated early plant growth and stress response [25]. By comparison, their function in salt stress has been less attended to.

As the most widely cultivated cereal crop, rice is known for its sensitivity to salt stress [26]. Identifying related genes involved in salt tolerance and uncovering the molecular mechanisms in rice is crucial for developing saline-tolerant varieties. To explore the potential role of *ABCG* subfamily genes in rice under salt stress, we analyzed the characteristics of the *ABCG* family, including phylogenetic relationships, gene structure, cis-acting elements, and evolution both within and between species. We investigated the expression levels of *ABCG* genes under salt stress to select candidate genes with potential for salt tolerance. The knockout mutant of the selected *ABCG* candidate gene was then generated to evaluate the potential roles under salt tolerance. Our study contributes to understanding the potential of *ABCG* genes in rice under salt stress, ultimately assisting in the cultivation of salt-tolerant rice.

## 2. Results

### 2.1. Identification of Rice OsABCGs, Phylogenetic and Evolutionary Analysis

**Figure 1:**
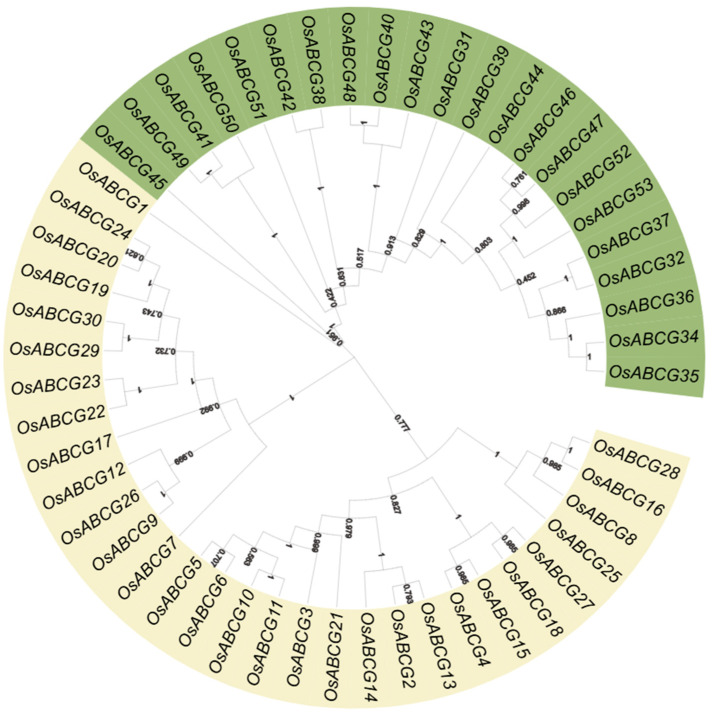
Phylogenetic analysis of the ABCG transporters among rice. The phylogenetic tree was derived with the Neighbor-joining (NJ) method in MEGA11. WBC and PDR represent two subgroups.

A phylogenetic tree was created to analyze the evolutionary relationships between diverse members of the *ABCG* gene family using 52 ABCG proteins from rice (Figure 1); *OsABCG33* has been classified as a pseudogene [19]. ABCG protein sequences were downloaded from the UniProt database (https://www.uniprot.org, accessed on 1 March 2024), Using the MEGA11 program to construct the phylogenetic tree based on the multiple alignments of all the ABCG protein sequences in rice species. ABCG members were found to have around 479–1479 amino acids (Supplementary Table S1). All the identified ABCGs were classified into two subgroups in the phylogenetic tree. Subgroup 1 (WBC) comprises 30 half-size ABCGs, while Subgroup 2 (PDR) consists of 22 full-size ABCGs.

### 2.2. Gene Structure Analysis of ABCGs

**Figure 2:**
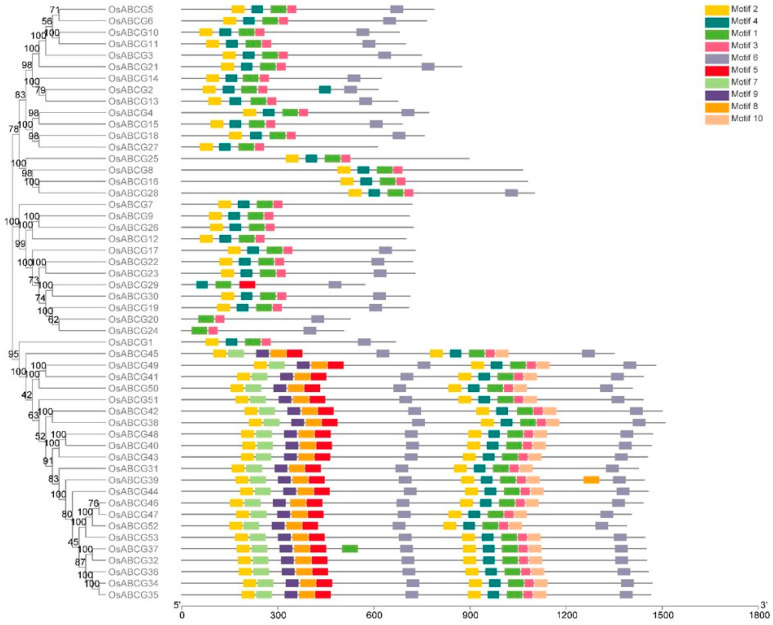
Phylogenetic relationships and protein motif analysis of OsABCGs. Ten conserved motifs are represented by different colors.

With the purpose of better understanding the similarity and diversity of the ABCGs, the conserved motifs of ABCG proteins were investigated (Figure 2). The MEME online tool (http://meme-suite.org/, accessed on 15 March 2024) was used to predict the conserved motif compositions. Motif prediction identified 10 conserved motifs. Motif1 was highly conserved in almost all ABCG proteins and belongs to the NBD domain, indicating a high degree of sequence conservation within the NBD sequences of ABCG proteins. In addition, each group had some degree of motif specificity. For example, Motif7, Motif8, Motif9, and Motif10 only existed in PDRs, indicating that they have subclade-specific functions. We found that members with relatively similar genetic relationships possess more similar motifs, indicating that ABCG members clustered in the same subgroup may have more similar biological effects.

### 2.3. Chromosomal Location and Gene Duplication Analysis of OsABCGs

**Figure 3:**
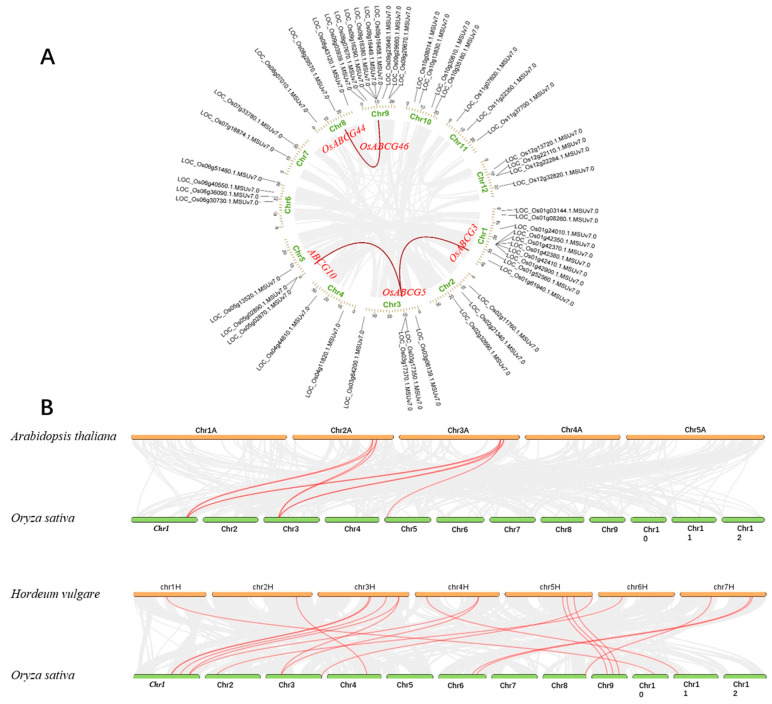
Chromosomal location and gene duplication of *OsABCGs* in the rice genome. (**A**) The duplicated gene pairs were connected by curved lines. (**B**) Synteny relationships analysis of *OsABCGs* between *Oryza sativa* and *Arabidopsis thaliana*, *Hordeum vulgare*. Gray lines indicate the collinear blocks, and the red lines indicate collinear gene pairs.

To further explore the relationships among the *OsABCG* genes, we performed a collinearity analysis of replication events within the *ABCG* family. According to Figure 3 there are a total of 52 genes distributed across all chromosomes. The highest number of genes is found on chromosomes 1 and 9, with 10 and 9 genes, respectively. Chromosomes 4, 7, and 8 only have two genes each (Figure 3A). We found three gene pairs (*OsABCG3/5*, *OsABCG5/10*, *OsABCG44/46*) formed three segmental duplication events on chromosomes 1, 3, 5, 8, 9, and 12 using the MCScanX method (Figure 3A) (Supplementary Table S2). We speculate that the degree of evolution of *OsABCGs* is relatively conservative within the species. To further understand the replication event of the *OsABCG* genes, the replication events of ABCG were compared between Rice and other species (*Arabidopsis thaliana* and *Hordeum vulgare*) (Figure 3B) (Supplementary Table S3). Collinearity analysis showed that the homologous genes between rice and *Arabidopsis thaliana* were 10 pairs, 18 homologous gene pairs between rice and *Hordeum vulgare*. *OsABCG* genes and *HvABCG* genes are similar, which is significant for exploring the relationships among species and forecasting gene function.

### 2.4. Analysis of ABCGs Promoter

**Figure 4:**
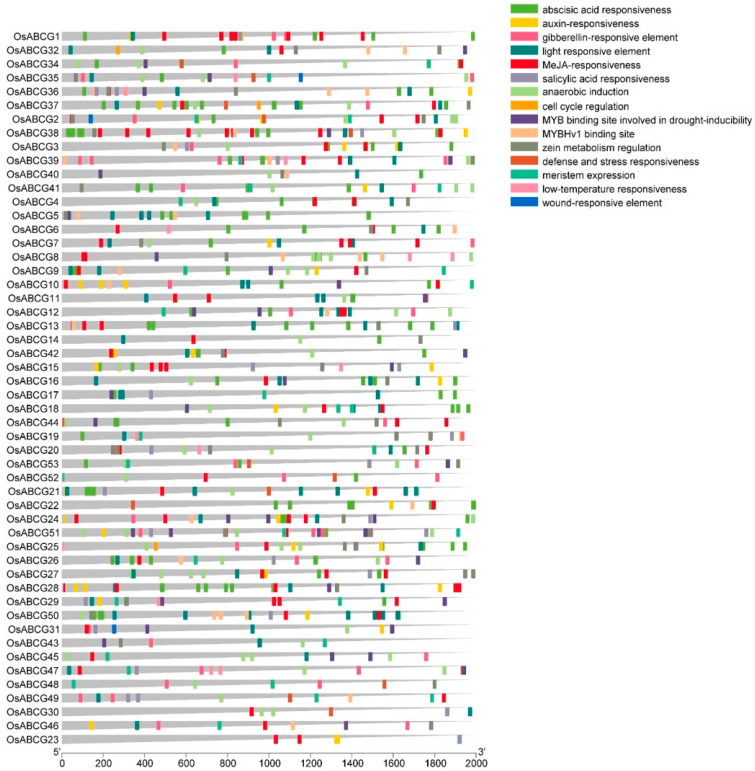
Cis-element analysis of *ABCG* genes. Each cis-acting is represented by a different color, and its position is the same as the corresponding position of the promoter.

To better understand the function and regulation of *ABCG* genes, the promoter sequences of all genes were analyzed by using PlantCARE (Supplementary Table S4). We found a series of cis-elements related to the abiotic stress response, plant hormone response, and plant growth and development were identified (Figure 4). There were light-responsive elements, anaerobic induction, MYB binding site involved in drought-inducibility, MYBHv1 binding site, defense and stress responsiveness, low-temperature responsiveness, wound-responsive element, and hormone-responsive elements, including the abscisic acid responsiveness, auxin-responsiveness, gibberellin-responsive element, MeJA-responsiveness, and salicylic acid responsiveness. In addition, some elements are involved in plant growth and development (zein metabolism regulation and meristem expression).

### 2.5. Expression Pattern Analysis of Rice ABCG Family Members under Salt Stress

**Figure 5:**
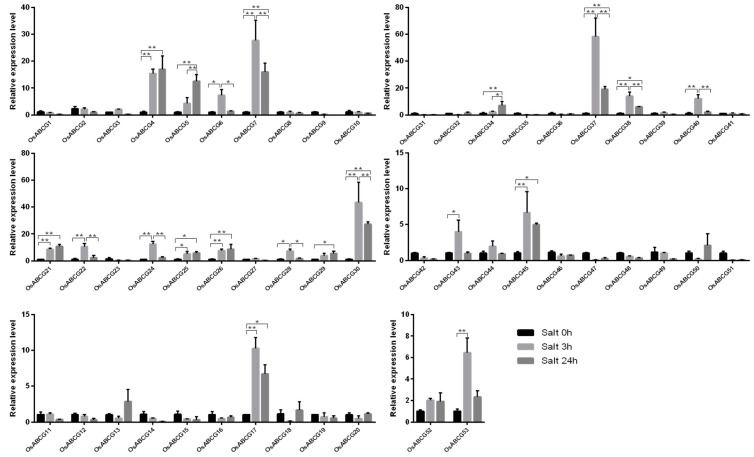
The relation expression of the rice *ABCG* genes under salt stress. Error bars represent the means ± standard deviation (SD) of three independent biological replicates, which were statistically analyzed using one-way ANOVA with the Tukey HSD test. * *p* < 0.05, ** *p* < 0.01.

To explore the expression level of the *ABCG* gene family under salt stress. The leaves of seedlings were selected and transplanted into the culture environments without or with 140 mm NaCl in the Yoshida medium, respectively. According to the q-PCR data, the expression levels of different genes under salt stress were analyzed (Figure 5). Under salt stress, the expression levels of 19 genes as *OsABCG4*, *OsABCG5*, *OsABCG6*, *OsABCG7*, *OsABCG17*, *OsABCG21*, *OsABC22*, *OsABCG24*, *OsABCG25*, *OsABCG26*, *OsABCG28*, *OsABCG29*, *OsABCG30*, *OsABCG34*, *OsABCG37*, *OsABCG38*, *OsABCG40*, *OsABCG45*, *OsABCG53* have shown significant differences. Expression of *OsABCG5*, *OsABCG21*, *OsABCG25*, *OsABCG29*, and *OsABCG34* genes continued to increase with increasing salt treatment time. The expression of *OsABCG4*, *OsABCG6*, *OsABCG7*, *OsABCG17*, *OsABCG22*, *OsABCG24*, *OsABCG28*, *OsABCG30*, *OsABCG37*, *OsABCG38*, *OsABCG40*, *OsABCG43*, *OsABCG45*, and *OsABCG53* genes was significantly increased at 3 h and decreased at 24 h (Figure 5). These *ABCG* genes may play a crucial role in the response to salt stress.

### 2.6. Perform Salt Tolerance Screening in Yeast

**Figure 6:**
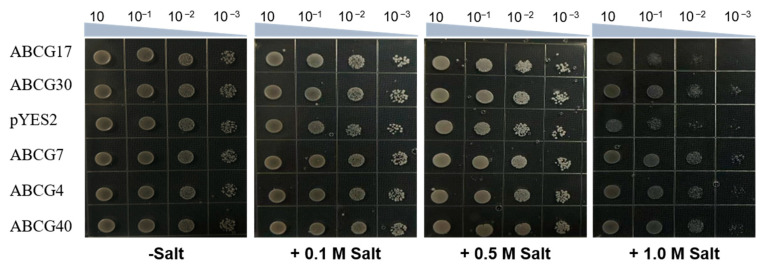
Yeast experiments with ABCGs. The growth conditions of recombinant yeast INVSc1 (*pYES2-ABCGS*) and control yeast INVSc1 (*pYES2*) with 0.1 M, 0.5 M, and 1.0 M NaCl. Cell density was adjusted to OD−600 at 1.0, and then we performed a serial dilution—spot 5 µL of each dilution at each point. The plates were photographed after 72 h of incubation at 30 °C.

Based on the q-PCR results, five candidate genes of interest were selected to analyze their salt resistance ability in yeast (Figure 6). The *ABCG4*, *ABCG7*, *ABCG17*, *ABCG30*, and *ABCG40* transformants, as well as the empty vector (*pYES2*), were individually plated on a culture medium without or with 0.1 M, 0.5 M, and 1 M NaCl, and their growth was observed. The results showed that in the absence of NaCl in the medium, there was no significant difference in growth between the empty vector and the yeast transformants carrying the candidate genes. However, when the NaCl concentration in the medium was 1M, there was a noticeable difference in growth between the *ABCG4*, *ABCG7*, *ABCG30*, and *ABCG40* transformants compared to the empty vector (*pYES2*), with the growth of the empty vector being more severely inhibited. The transformants carrying *ABCG7* exhibited stronger growth compared to the other transformants.

### 2.7. Mutations of OsABCG7 Exhibits a Salt-Sensitive Phenotype

**Figure 7:**
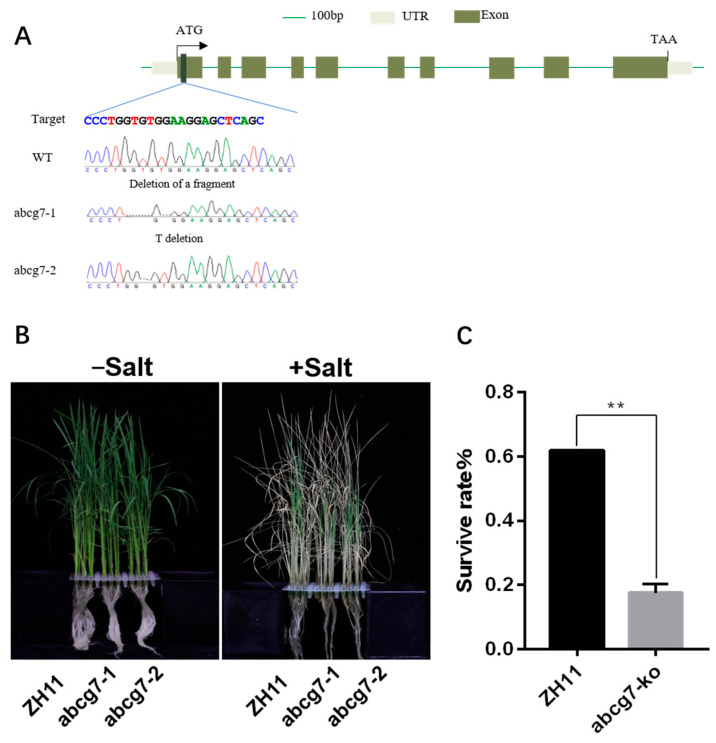
CRISPR/Cas9-induced mutation in the *OsABCG7* gene. (**A**) Schematic diagram of gene structure and one CRISPR/Cas9 target locations, the 20-nt target sequences are shown at the bottom of the gene structure; (**B**) The phenotypic comparison of the abcg7 and WT; (**C**) Survival rate statistics of ZH11 and abcg7-ko after rehydration. The values shown in (**C**) are means ± SD (*n* = 20–24 plant for each repeat) of three biological replicates, which were statistically analyzed using one-way ANOVA with the Tukey HSD test. ** *p* < 0.01.

To further confirm the role of *OsABCG7* in salt stress, we obtained two lines of the abcg7 knockout mutant generated with the CRISPR/Cas9 system. Two homozygous mutants (abcg7-1 with a fragment deletion in target and abcg7-2 with a T deletion in target) were obtained by sequencing (Figure 7A). Ten-day-old seedlings of abcg7 mutants and wild type (ZH11) were treated with 140 mm NaCl for 7 days. The results showed that no phenotypic differences were observed under normal growth conditions. However, under salt stress, the mutant displayed a more pronounced wilting phenotype than ZH11. (Figure 7B). One week after rehydration, the survival rate of the mutant was significantly lower than that of the wild-type ZH11 (Figure 7C), indicating a salt-sensitive phenotype.

## 3. Discussion

In plants, the ABCG protein subfamily is notably abundant and plays a crucial role in diverse signaling pathways throughout plant development and stress responses [27,28,29]. Despite this, the specific functions of ABCG proteins under salt stress conditions have been comparatively less explored. To address this gap, we have undertaken a comprehensive analysis of *ABCG* genes, offering valuable insights into their evolutionary trajectory and functional attributes.

The phylogenetic tree describes the evolutionary relationships between organisms and is crucial for studying the evolution of life [30]. In this study, a phylogenetic tree was constructed for 52 *ABCG* genes in rice, and the conserved regions of these 52 genes were analyzed. The results show that these genes are mainly divided into two subgroups: *ABCG1* to *ABCG30* are half-size proteins (WBC), each containing at least three conserved motifs, whereas *ABCG31* to *ABCG53* are full-size proteins (PDR), each containing ten conserved motifs, high structural similarity among genes within the same subgroup, this similarity may be due to similar motifs.

Paralogous gene pairs refer to genes within the same species that have originated from gene duplication events and may have evolved new but related functions to the original. Orthologous gene pairs are genes from different species that have evolved from a common ancestral gene through speciation and typically maintain the same function over time. Identifying paralogous and orthologous genes can provide a better understanding of the key processes of gene function [31]. Unsurprisingly, we found three duplicated gene pairs were classified under the same subfamilies in the phylogenetic tree such as *OsABCG3*, *OsABCG5*, and *OsABCG10* from WBC-like, *OsABCG44*, and *OsABCG46* from PDR-like subfamilies. Additionally, we hypothesize that the *ABCG* genes have shown diversity during the evolutionary process in rice, allowing them to develop normally under constantly changing environmental conditions. We extracted ABCG family members from the salt-sensitive crop Arabidopsis and the salt-tolerant crop barley for interspecies collinearity analysis [32]. The findings suggest that rice has a closer relationship with barley, suggesting that rice may possess potential salt tolerance.

When plants experience stress, activated transcription factors bind to cis-acting elements, leading to the activation of target gene promoters and the expression of genes involved in stress resistance [33,34]. This study found that the cis-acting elements of ABCGs are mainly associated with responses to abiotic stress, hormone signaling, and growth development. ABCG7 contained four MeJA-responsiveness: under salt stress, methyl jasmonate (MeJA) treatment can regulate the growth and developmental activities of plants [35]. These findings suggest that ABCGs play important roles in abiotic stress tolerance and growth and developmental processes.

Gene expression is an important part of the transmission and expression of genetic information in organisms. Analyzing gene expression patterns helps in identifying the pathways and regulatory mechanisms of gene expression. Our qPCR data indicate that the expression levels of 19 genes have been upregulated in the salt stress environment, which is consistent with the trends observed in previous studies [15,19].

We selected some candidate genes that were subjected to yeast salt tolerance assays, and it was found that the growth of transformants with *ABCG4*, *ABCG7*, *ABCG30*, and *ABCG40* was stronger than that of the empty vector *pYES2*. It is speculated that these genes may be related to the salt tolerance of rice.

To explore the regulatory mechanism of the *OsABCG7* gene under salt stress, we utilized the CRISPR-Cas9 technology to knock out the *OsABCG7* gene in the rice variety ZH 11, resulting in two mutant lines, abcg7-1 and abcg7-2. The results showed that under salt stress, the growth vigor of the abcg7-1 and abcg7-2 was weaker than that of the wild type, and the survival rate was significantly reduced, exhibiting a salt-sensitive phenotype. This study is significant for further research on the ABCG family in rice in response to salt stress conditions, which may improve salt tolerance and increase the yield of rice.

## 4. Materials and Methods

### 4.1. Identification of Rice OsABCGs Phylogenetic and Evolutionary Analysis

To perform phylogenomic analysis of ABCG transporters in rice, we performed keyword ABCG at UniProt (https://www.uniprot.org/ accessed on 1 March 2024) database for related genes to look for any additional member and performed a BLASTP against the Phytozome databases (https://phytozome-next.jgi.doe.gov/ accessed on 1 March 2024). Multiple sequence alignments were performed using Clustal W, the phylogenetic tree of ABCG amino acid sequences in Rice was constructed with MEGA 11 by the NJ method with 1000 bootstrap value [36,37], and the phylogenetic tree was visualized through EvolView (https://www.evolgenius.info/evolview/ accessed on 2 March 2024).

### 4.2. Analysis of Conserved Protein Motifs of OsABCGs

To examine the characteristics and properties of the rice ABCG subfamily protein domain, the web-based tool Motif Elicitation (MEME) (http://meme-suite.org/tools/meme, accessed on 5 March 2024) was adopted for detecting conserved motifs within ABCG proteins. The conserved motif of OsABCGs was displayed in TBtools.

### 4.3. Analysis of Chromosomal Location and Gene Duplication

The *Oryza sativa*, *Arabidopsis*, and *Hordeum vulgare* genomic feature files (gff3 and fasta) were downloaded from the Phytozome (https://phytozome-next.jgi.doe.gov, accessed on 5 March 2024), and the chromosomal location of each gene was plotted using TBtools software. The syntenic connections between *ABCG* genes were determined using the MCScanX module of TBtools, and Circos and collinearity diagrams were drawn using the Circos and Synteny plot module in TBtools to visualize the gene density and the collinearity [38].

### 4.4. Analysis of Cis-Elements of OsABCG Genes

The upstream 2000 bp genome sequence was extracted and defined as the promoter using the fasta extract module in Tbtools. The *OsABCG* regulatory elements in gene promoter regions were made using the PlantCARE website (http://bioinformatics.psb.ugent.be/webtools/plantcare/html, accessed on 8 March 2024). The detected elements were divided into different response types based on their annotated functions.

### 4.5. RNA Isolation and Quantitative Real-Time PCR Analysis under Salt Stress

We selected 52 genes containing elements that responded to adversity for salt stress analysis based on the above analysis of cis-elements in the promoter region. Total RNA was extracted in leaves of seedlings treated with 140 mM NaCl solution for 0, 3, and 24 h (Trizol reagent kit, TransGen Biotech, Beijing, China). Next, 1 µg of total RNA was separated via 1% agarose gel electrophoresis to assess its quality and integrity. The RNA concentration and purity were measured with a spectrophotometer (NANO-300, ALLSHENG, Zhejiang, China). The residual DNA was treated with RNase-free DNase I, and then the first strand of cDNA was synthesized using HisyGo RT Red SuperMix for qPCR kit (Vazyme, Nanjing, China) following the manufacturer’s instructions. After reverse transcription, the reaction product was diluted 5-fold with sterile water as backup. Primers specific for the 52 *OsABCG* genes were designed with SnapGene software (www.snapgene.com, accessed on 1 May 2024). Subsequent q-PCR analysis was performed on a LightCycler 480 II system using the SYBR Green PCR Master Mix (TransGen Biotech, Beijing, China). The gene primers selected were synthesized on the Tsingke Biotech (Beijing Tsingke Biotech Co., Ltd.) and are shown in Supplementary Table S5. Relative transcript levels and fold change were calculated by the 2^−∆∆Ct^ method, respectively [39]. All analyses were performed with three biological replicates, and q-PCR data were analyzed using IBM SPSS 23 software (Armonk, NY, USA: IBM Corp).

### 4.6. Transport Assays in Yeast

Connect the candidate *ABCGs* gene coding sequences (CDS) to the yeast expression vector *pYES2* using SnapGene software (Primers were listed in Supplementary Table S5). Verify their correctness through sequencing. Transform the empty vector *pYES2* and *pYES2-ABCGs* (*ABCG4*, *ABCG7*, *ABCG17*, *ABCG30*, *ABCG40*) into INVSc1 cells. After 2 days of cultivation in SD-Ura medium, perform colony PCR verification. Inoculate single colonies carrying *pYES2* and *pYES2-ABCGs* into 6 mL SD-Ura liquid medium and cultivate at 30 °C and 220 rpm for 16 h. Centrifuge at 12,000× *g* for 5 min at room temperature, discard the supernatant and resuspend the cells in an appropriate amount of sterile water to achieve an OD600 of 0.8–1.0. Make 1:10 gradient dilutions of the bacterial suspension and spot 5 μL of each dilution onto SC/-Ura medium supplemented with 0 M, 0.1 M, 0.5 M, and 1.0 M NaCl concentrations. Incubate inverted at 30 °C for 3 days, observe the results and record photographs.

### 4.7. Plant Materials and Treatments

The abcg7 mutant was generated by CRISPR/Cas9 using the vector pRGEB32 (wimibio, Jiangsu, China). Agrobacterium tumefaciens (strain EHA105) mediated transformation was used to introduce the constructs into rice [40]. Positive lines were confirmed by PCR and sequencing, and then the homozygous T_2_ plants were used for salt treatment experiments. Primers used for PCR amplification and vector construction were listed in Supplementary Table S5. Rice seeds were soaked in water for 2 days, and then the germinated seeds were put on a net floating on Yoshida solution in a greenhouse (16/8 h light-dark cycles at 28 °C) and used for various experiments. For the salt treatment, 12-day-old seedlings were exposed to 140 mM concentration of NaCl for 7 days. For each condition, shoots from 3 to 5 plants were collected at every time point for RNA extraction.

### 4.8. Statistical Analysis

Data were analyzed using GraphPad Prism version 7.0 for Windows (GraphPad version 7.0 Software, Boston, MA, USA, accessed on 25 May 2024). One-way ANOVA was performed to determine the significance of differences between treatments. *p* < 0.05 (Tukey’s test) was considered significant.

## 5. Conclusions

The *ABCG* gene family plays an important role in both plant growth and stress resistance. In this study, we systematically analyzed the *ABCG* gene family using bioinformatics tools, including phylogenetic tree analysis, gene structure and conserved motif identification, chromosomal mapping, cis-element prediction, and the expression changes of each gene under salt stress. Our q-PCR results demonstrated that some *ABCG* genes are induced by salt stress, and we efficiently and rapidly identified a candidate gene, *OsABCG7*, associated with salt stress response. Although the function of *ABCG* genes in salt stress response remains to be elucidated, our results provide valuable insights into the important roles played by members of the ABCG subfamily in the adaptive mechanisms of plant responses to salt stress, and this information is crucial for strategies to improve salt tolerance in rice.

## Data Availability

Data are contained within the article.

## Supplementary Materials

The following supporting information can be downloaded at https://www.mdpi.com/article/10.3390/ijms251910724/s1.
